# Exploiting Signaling Pathways and Immune Targets Beyond the Standard of Care for Ewing Sarcoma

**DOI:** 10.3389/fonc.2019.00537

**Published:** 2019-06-19

**Authors:** Dana L. Casey, Tsung-Yi Lin, Nai-Kong V. Cheung

**Affiliations:** ^1^Department of Radiation Oncology, Memorial Sloan Kettering Cancer Center, New York, NY, United States; ^2^Department of Pediatrics, Memorial Sloan Kettering Cancer Center, New York, NY, United States

**Keywords:** Ewing sarcoma, antibodies, immunotherapy, targeted therapy, pediatric sarcomas

## Abstract

Ewing sarcoma (ES) family of tumors includes bone and soft tissue tumors that are often characterized by a specific translocation between chromosome 11 and 22, resulting in the EWS-FLI1 fusion gene. With the advent of multi-modality treatment including cytotoxic chemotherapy, surgery, and radiation therapy, the prognosis for patients with ES has substantially improved. However, a therapeutic plateau is now reached for both localized and metastatic disease over the last two decades. Burdened by the toxicity limits associated with the current frontline systemic therapy, there is an urgent need for novel targeted therapeutic strategies. In this review, we discuss the current treatment paradigm of ES, and explore preclinical evidence and emerging treatments directed at tumor signaling pathways and immune targets.

## Introduction

Ewing sarcoma (ES) family of tumors is a family of small round blue cell tumors that arise from bone or soft tissue. It represents the second most common malignant bone tumor in children and young adults, with an incidence of more than 200 cases per year in the United States (1). ES is characterized by a specific translocation involving EWS (Ewing sarcoma gene) on chromosome 22 with one of the E26 transformation-specific transcription factory family genes. The EWS-FLI1 (Friend Leukemia Integration 1 transcription factor) fusion gene, t(11;22)(q24;q12) is found in ~85% of ES tumors. The fusion protein plays a key role in the pathogenesis and proliferation of ES (2, 3), with EWS-FLI1 knockdown cells showing decreased proliferation *in vitro* and tumor regression *in vivo* (4, 5). Although the fusion protein has multiple functions, one of its primary roles is as a transcription factor, increasing the expression of many downstream targets involved in tumor survival and growth [for example, *IGF1* (6), *GLI1* (7), *Myc* (8), *ID2* (9)], while decreasing expression of cell cycle regulators and pro-apoptotic genes [for example, *TGFB2* (10), *p21* (11), *IGFBP3* (12)]. In addition, the fusion protein plays an important role in promoting cell differentiation by upregulating such genes as *EZH2* (13) and *SOX2* (14). Although ES cells were originally thought to arise from primitive neuroectodermal cells, there is now growing evidence (although not conclusive) that ES cells arise instead from mesenchymal stem cells (15, 16), and that the neuroectodermal phenotype of ES is secondary to EWS-FLI1 expression (17).

With the introduction of multi-disciplinary management and specifically cytotoxic chemotherapy, survival for localized ES has improved from <20 to 70–80% by the 1990's. However, over the last two decades, there has been no further advancement in survival, witnessing the limit of further intensification of cytotoxic chemotherapy to cure children and young adults with localized disease. Additionally, the current frontline systemic therapy is aggressive and carries with it significant morbidity. For patients with metastatic disease, prognosis has remained poor, with survival rates of <30% in those with isolated lung metastases and <20% for those with bone and bone marrow involvement (18, 19). Outcomes for patients with relapsed disease is even poorer, with a 5-year survival rate of only 13%. Given these considerations of toxicity and suboptimal survival from metastatic disease, there is an urgent unmet need to develop novel therapies for ES (20).

Molecularly targeted therapy and immunotherapy are promising approaches for attacking these tumors without a significant increase in overlapping toxicity with chemoradiation (21, 22). A good example of the potential for immunotherapy in children is the use of anti-GD2 antibody in metastatic high risk neuroblastoma where cures beyond 10 years are now possible in the majority of patients without appreciable late effects from the anti-GD2 antibody (23, 24). Although the EWS-FLI1 fusion protein is present only in ES tumor cells and not in normal tissue (providing an ideal target for drug development), EWS-FLI1 targeted therapy has so far been unsuccessful in the clinic. In this review, we summarize the current treatment paradigm of ES, and emerging therapies for ES, including molecularly targeted therapy and immunotherapy.

## Frontline Therapy

### Localized Disease

Although <25% of patients present with gross metastatic disease, ES is considered a systemic disease with subclinical spread (25). In fact, patients with ES who undergo local therapy alone experience relapse rates approaching 90% (26). Thus, the current treatment paradigm for ES consists of multimodality therapy with chemotherapy, surgery, and/or radiation therapy (RT). Chemotherapy is considered the backbone of therapy for ES, and is typically given both neoadjuvantly and adjuvantly. Induction therapy is specifically recommended for ES to address micrometastatic disease as well as to reduce the size of the tumor, potentially allowing for a less extensive or less morbid surgery (and/or smaller radiation volumes).

The first two Intergroup Ewing sarcoma studies (IESS) established the use of vincristine, doxorubicin, cyclophosphamide, and actinomycin A (VDCA) with dose-intensive doxorubicin as the standard of care (27, 28). IESS-III was a phase III randomized clinical trial that showed a relapse-free survival benefit with the addition of ifosfamide and etoposide to VDCA (18). Subsequent trials omitted actinomycin D with no deleterious effect on outcomes. Given these findings, standard chemotherapy for ES now consists of vincristine, doxorubicin, and cyclophosphamide, with the addition of ifosfamide and etoposide (VDC/IE). Although dose intensification of the alkylating agents did not improve outcomes for patients with localized ES in a large Children's Oncology Group (COG) study (29), interval compressed-therapy (cycles given every 14 days rather than every 21 days) did result in improved outcomes (30). As such, interval-compressed chemotherapy with alternating cycles of VDC/IE is recommended as first-line systemic therapy for localized disease.

Surgical resection and/or RT are done for local control of the primary tumor, with considerations given to the location of the tumor, the response to induction therapy, and the degree of morbidity associated with resection vs. RT. Typically, for patients with ES arising from dispensable bones (i.e., fibula, ribs) or from the axial skeleton in which a margin-negative resection can be performed without excessive morbidity, surgical resection is the preferred choice of local control, sparing children the risk of second malignant neoplasms after RT. However, for patients with tumors arising from the pelvis, spine, or other locations in which function-preserving (or limb-sparing), margin-negative surgery cannot be performed, definitive radiation is the preferred modality of treatment. There are no randomized trials directly comparing the efficacy of surgery vs. RT for local control, but some retrospective studies and a systematic review have shown that local control may be superior with surgery (31–33). However, retrospective studies can be flawed because of selection bias, as tumors that are easily surgically resectable are often smaller tumors in more favorable locations.

### Metastatic Disease

For patients with metastatic disease, outcomes remain poor and are dependent on the site of metastatic disease as described above, with the worst prognosis for those that spread to bone and bone marrow. Systemic chemotherapy used for patients who present with metastatic disease is similar to that used for patients with localized disease. However, unlike patients with localized disease in which the addition of ifosfamide and etoposide to vincristine, doxorubicin, and cyclophosphamide has been shown to improve survival, the addition of ifosfamide and etoposide has not shown benefit for those with metastatic disease (18, 34). Despite these concerns, front-line treatment for patients with metastatic disease still employs VDC/IE. While dose-intensified VDC/IE with augmented alkylator dose does not seem to improve outcomes for patients with metastatic disease (35), the role of interval-compressed chemotherapy remains under investigation on COG AEWS1221 (NCT02306161). The role of high-dose chemotherapy with hematopoietic cell support for patients with metastatic disease has also been explored. Some studies have shown excellent event-free survival rates in patients who received high-dose chemotherapy followed by autologous transplant (36), while others have shown no benefit (37, 38). The role of autologous stem cell rescue was further explored on the EURO-EWING 99 trial, with interim results showing no improvement in survival with stem cell rescue for patients with metastatic disease to the lung compared to conventional chemotherapy with whole lung irradiation (39). Of note, stem cell rescue on this trial did improve survival for patients with localized disease at high risk of relapse, though (40). Given different inclusion criteria and stratification approaches, it is difficult to compare these results to those from the COG showing a benefit of interval-compressed chemotherapy in the localized setting. However, the outcomes appear similar after either dose-intensification approach (stem-cell transplant vs. intensively timed chemotherapy), and there is no consensus across continents regarding front-line approach.

Treatment of metastatic sites of disease with radiation and sometimes even surgery has been considered as part of consolidation. For example, for patients with pulmonary metastases, non-randomized studies supported the delivery of whole lung irradiation after completion of chemotherapy; (41) and for patients with residual lung metastases after completion of chemotherapy, further surgical resection has been advocated (42). Consideration has also been given to irradiating bone and soft tissue metastases, especially in the setting of oligometastatic disease. On the ongoing COG metastatic ES protocol, AEWS1221, patients can be treated with stereotactic body RT to up to five sites of metastatic disease. Local control of the primary site is also important for patients with metastatic disease, with RT often preferred over surgical resection unless there has been a significant response to chemotherapy.

### Relapsed Disease

As is true for patients with metastatic disease, the prognosis of patients with local relapse remains poor. Phase II clinical trials have shown activity of camptothecin-based approaches with either topotecan or irinotecan for recurrent disease (43–45). Other agents utilized in the recurrent setting such as gemcitabine, docetaxel, bortezomib, and ecteinascidin-743 have not improved survival (46–49). Given the dose-limiting toxicities of cytotoxic chemotherapy and the overall poor outcomes, patients with recurrent ES should be considered for clinical trials using novel molecularly targeted therapies or immune-based approaches as discussed in more detail below.

## Molecularly Targeted Therapy (Figure 1)

### EWS-FLI1 Pathway (Clinical Data)

The EWS-FLI1 fusion protein results in the production of a unique tumor driver only found in tumor cells. Given this specificity, targeted therapy directed at the EWS-FLI1 protein should avoid non-specific toxicities. Additionally, tumorigenesis in ES is dependent on EWS-FLI1 fusion protein expression, with deletion resulting in ES cell death in pre-clinical studies (5, 50, 51). This mechanistic dependency on the fusion protein further supports EWS-FL11 as an obvious therapeutic target. Despite these considerations, there is no available drug that can directly inhibit the fusion protein. Studies in the 1960's and 1970's utilizing various peptides and natural products to target the EWS-FL1I fusion protein showed activity in the preclinical setting (Table 1), although their translation into the clinical setting was limited by toxicity. For example, mithramycin is a natural product known to repress the EWS-FLI1 protein *in vitro*. A phase I/II study including eight patients with refractory ES treated with mithramycin showed no clinical responses with an inability to safely achieved the desired dose secondary to hepatotoxicity (63).

**Figure 1:**
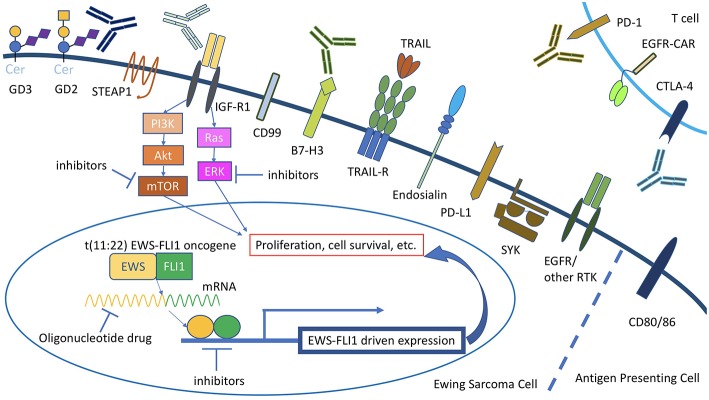
Overview of current molecularly targeted therapy for Ewing sarcoma. For STEAP1 and CD99, antibodies have been tested in mouse models of Ewing sarcoma but not in the clinical trial setting. Cer, ceramide.

**Table 1:** Preclinical studies targeting the EWS-FLI1 pathway.

**Drug**	**Drug type**	**Molecular target**	**Mechanism**	**References**
EC-8042 and EC-8105	Chemicals (natural product, mithramycin analogs)	EWS-FLI1 protein	Represses EWS-FLI1 activity by decreasing expression of EWS-FLI1 downstream targets	(52)
Englerin A	Chemicals (natural product)	EWS-FLI1 protein	Inhibits cellular proliferation through a decrease in EWS-FLI1 phosphorylation and reduction its DNA binding ability	(53)
ESAP1 (TMRGKKKRTRAN)	Chemicals (synthetic peptide)	EWS-FLI1 protein	Impairs the transcriptional activity of EWS-FLI1 and blocks cell cycle progression	(54)
Romidepsin, Depsipeptide, FK228, entinostat (MS-27-275)	Chemicals (natural product, synthetic peptide)	Histone deacetylase	Reverses EWS-FLI1 mediated histone deacetylation, decreases *EWS-FLI1* mRNA and protein levels, inhibits cell proliferation, and induces TRAIL-dependent apoptosis of ES cells	(55–57)
LSD1 inhibitor HCI-2509	Chemicals (benzoic hydrazide)	Lysine specific demethylase 1 (Histone demethylase)	Comprehensively reverses the transcriptional profiles driven by both EWS-FLI and EWS-ERG, and markedly delays tumorigenesis *in vivo*	(58)
JIB-04	Chemicals (pyridine hydrazone)	Jumonji domain containing histone demethylases	Deregulates oncogenic programs and increases DNA damage, resulting in impaired cell proliferation and survival, and reduced tumor growth	(59)
Arsenic trioxide	Chemicals (inorganic arsenic compound)	Glioma-Associated Oncogene Homolog 1 (GLI1)	Inhibits ES tumor growth via the inhibition of GLI1	(60)
Methylseleninic acid (MSA)	Chemicals (organic selenium compounds)	FOXO1, Forkhead box family protein	Increases FOXO1 expression in the presence of EWS-FLI1, induces massive cell death and decreases xenograft tumor growth dependent on FOXO1	(61, 62)

Strategies to inactivate or decrease the expression or function of the EWS-FLI1 protein have shown some promise, including inhibitory oligonucleotides and small-molecule inhibitors that are able to disrupt its transcriptional complex. Inhibitory oligonucleotides are short nucleotide sequences that can be designed to hybridize to single-stranded mRNA molecules and subsequently inhibit protein translation (64). Both antisense oligonucleotides and inhibitory RNA can be utilized for this purpose. For ES, inhibitory oligonucleotides have been designed that can bind to selected sequences coding for the EWS-FLI1 fusion protein, consequently decreasing expression of the fusion protein and resulting in decreased tumor growth in preclinical models (65, 66). Although inhibitory oligonucleotides have been successfully used to treat ES *in vitro*, translation to humans has proven difficult partly because of the inefficiency of drug transport intracellularly for maximal activity (67). Thus, inhibitory oligonucleotides for ES are not currently in the clinic.

The Toretsky lab at Georgetown has pioneered the inhibition of the EWS-FLI1 protein via disruption of protein-protein interactions. Specifically, they have created a peptide that competes with wild type RNA helicase A for a specific binding site on the EWS-FLI1 protein. This interaction between RNA helicase A and EWS-FLI1 is necessary for the function of EWS-FLI1 (68, 69). The small-molecule inhibitor of RNA helicase A, YK-4-279, has shown activity against ES *in vitro* (70), and an analog of YK-4-279, TK216, is currently being tested in a Phase 1 trial in relapsed or refractory ES (NCT02657005).

Poly (ADP-ribose) polymerase 1 (PARP1) is an enzyme involved in transcriptional regulation and DNA repair. PARP1 interacts with the EWS-FLI1 protein to create a positive feedback loop for transcriptional activation. Given the disruption of this critical interaction with PARP inhibitors, ES is highly responsive to PARP inhibition in preclinical models (71). Other studies have found that the concurrent administration of the PARP inhibitor, olaparib, with radiation results in increased lethal DNA damage and as a result, increased cell death *in vitro* (72). A phase II study including 12 patients with refractory ES showed that olaparib was well-tolerated, although no significant responses were seen, with patients progressing at a median time of 5.7 weeks from initiation of therapy (73). Given the preclinical efficacy data of combining olaparib with temozolomide (74), there is now an ongoing phase I study testing the safety and efficacy of this combination in patients with ES (NCT01858168).

### EWS-FLI1 Pathway (Preclinical Data)

Other approaches involve targeting downstream signaling molecules driven by the EWS-FLI1 fusion protein such as Glioma-Associated Oncogene Homolog 1 (GLI1). GLI1 is a transcription factor that is upregulated by EWS-FLI1 and plays an important role in the Hedgehog pathway (7). In ES specifically, when upregulated by EWS-FLI1, GLI1 plays a major role in maintaining the malignant phenotype and cell growth (75). In mice, antineoplastic arsenic trioxide (ATO) inhibits ES tumor growth via the inhibition of GLI1 (60). Anecdotal reports using a combination of ATO and standard chemotherapy (VP-16 and paclitaxel) have encountered minimal toxicities (76). A second candidate of the EWS-FLI1 pathway is the Forkhead box (FOX) gene family of proteins. The EWS-FLI1 gene regulates, though indirectly, the expression of FOXO1 which controls tumor growth and differentiation (61). In an orthotopic xenograft mouse model, methyl-imino selenium acid (MSA) was found to reactivate endogenous FOXO1, thereby significantly decreasing tumor growth (62). Unlike FOXO1, the FOXM1 protein is upregulated by EWS-FLI1 and serves as an oncogenic mediator that results in tumor proliferation; a reduction of FOXM1 protein results in decreased anchorage independent growth (77). Inhibition of FOXM1 can be achieved via thiostrepton both *in vitro* and *in vivo* (78), suggesting that FOXM1 may also serve as a potential therapeutic target of the FOX gene family. Tsafou et al. also performed an integrative drug screening analysis to identify mechanisms and compounds that interfere with the EWS-FL11 pathway and EWS-FL1 cell viability (79). Among the druggable targets identified, the authors found that MCL-1 (a known inhibitor of apoptosis) is directly activated by the fusion protein, suggesting a potential role of BLC-2 family inhibitors in ES.

### Insulin-Like Growth Factor (IGF) Pathway

The IGF family of ligands and receptors play key roles in normal human growth and development; not surprisingly, they have been implicated in various types of human cancers. Insulin-like growth factor 1 (IGF-1) is required for the growth of fibroblasts, epithelial cells, bone marrow stem cells, and osteoblasts (80). The binding of IGF-1 to its receptor, IGF-1R, initiates a cascade of events that affect protein turnover, exerting potent mitogenic and differentiating effects on most cell types. In preclinical models of ES, the IGF-1R-mediated signaling pathway is constantly active, suggesting its role in the tumorigenesis of ES (4, 81–85). As such, IGF-R is another attractive target for ES, where its inhibition both *in vitro* and *in vivo* have impaired the migratory ability of ES cells thereby slowing tumor growth (83, 86, 87).

**Figure 2:**
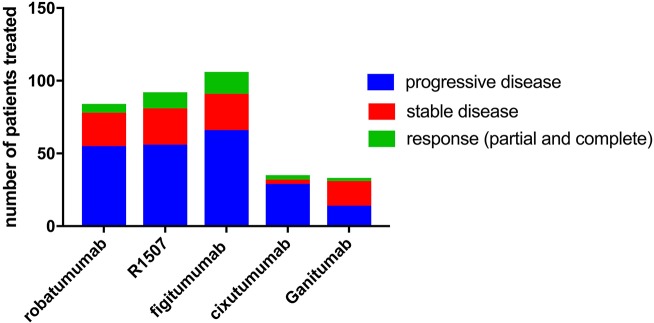
Clinical trial results of anti-IGF-1R therapy across phase II trials.

Both small molecule and antibody-mediated approaches to block the IGF pathway have been investigated in early phase clinical trials (Table 2) (89, 97, 108–110). Five human anti-IGF-1R antibodies for ES have been tested in phase II clinical trials: robatumumab (89) (also known as SCH 717454 and MK-7454, Merck and Schering-Plow), R1507 (111) (Roche), ganitumab (98) (NantCell, previously known as AMG 479 by Amgen Inc), cixutumumab (94, 95) (IMC-A12, ImClone systems), and figitumumab (93) (CP-751871, Pfizer). Among them, figitumumab showed the highest objective response rate of 14.2%; R1507 showed a response rate of 10.8%, cixutumumab 8.6%, robatumumab 7.1%, and ganitumab 6.1% (Figure 2). An ongoing phase III trial through the COG is testing the addition of ganitumab to combination chemotherapy in patients with newly diagnosed metastatic ES (NCT02306161). In addition, three other antibodies have been tested in the phase I setting: dalotuzumab (MK-0646) (99), BIIB022 (100), and AVE-1642 (101). In terms of their clinical efficacy, only one of six patients treated with dalotuzumab had a partial response; no patients treated with BIIB022 responded; and three of 40 patients treated with AVE-1642 had a partial response.

**Table 2:** Antibody-based approaches, immunotherapy, and small molecule inhibitors tested in clinical trials for Ewing sarcoma.

**Drug**	**Molecular target**	**Phase**	**Clinicaltrial.org identifier**	**Number of patients**	**Response rate (RR %)**	**References**
Mithramycin	EWS-FLI1 pathway	1/2	NCT01610570	8	0	(63)
TK216	EWS-FLI1 pathway	1	NCT02657005	45	N/A	Ongoing, recruiting
Olaparib	PARP1	2	NCT01583543	12	0	(88)
Olaparib + temozolomide	PARP1	1	NCT01858168	93	N/A	Ongoing, recruiting
Robatumumab	IGF-R1	2	NCT00617890	84	7.2	(89)
R1507	IGF-R1	1	NCT00560144	9	22.2	(90)
R1507	IGF-R1	2	NCT00642941	92	10.8	(91)
Figitumumab	IGF-R1	1	NCT00474760	16	12.5	(92)
Figitumumab	IGF-R1	2	NCT00560235	106	14.2	(93)
Cixutumumab	IGF-R1	1/2	NCT00668148	35	8.6	(94, 95)
Cixutumumab + Temsirolimus	IGF-R1 + mTOR	2	NCT01614795	46	0	(96)
Ganitumab	IGF-R1	1	NCT00562380	12	16.7	(97)
Ganitumab	IGF-R1	2	NCT00563680	33	6	(98)
Ganitumab + chemotherapy	IGF-R1	3	NCT02306161	330	N/A	Ongoing, recruiting
Dalotuzumab (MK-0646)	IGF-R1	1	NCT01431547	6	16	(99)
BIIB022	IGF-R1	1	NCT00555724	40	7.5	(100)
AVE1642	IGF-R1	1	UK study	40	8	(101)
Ipilimumab	CTLA4	1/2	NCT02304458	484	N/A	Ongoing, recruiting
Imatinib	c-KIT + PDGF-R	2	NCT00031915	185	1.6	(102)
Imatinib	c-KIT + PDGF-R	2	NCT00062205	7	14.2	(103)
Imatinib	c-KIT + PDGF-R	2	NCT00030667	70	1.7	(104)
Bevacizumab	VEGF-R	2	NCT00516295	7	N/A	Closed
Pazopanib	Multi-targeted RTK	1	NCT00929903	53	3.9	(105)
Lexatumumab	TRAIL-R	1	NCT00428272	24	0	(106)
Hu14.18K322A	GD2	1	NCT02159443	100	N/A	Ongoing, recruiting
Ontuxizumab	Endosialin	1	NCT01748721	27	0	(107)
Enoblituzumab	B7-H3	1	NCT02982941	25	N/A	Active, not recruiting
Nivolumab + ABI-009	PD1 + mTOR	1/2	NCT03190174	40	N/A	Ongoing, recruiting
Ipilimumab ± Nivolumab	CTLA4 ± PD1	1/2	NCT02304458	484	N/A	Ongoing, recruiting
Ipilimumab + Nivolumab	CTLA4+PD1	2	NCT02982486	60	N/A	Not yet recruiting
EGFR806 CAR T Cell	EGFR	1	NCT03618381	36	N/A	Ongoing, recruiting
Sarcoma-specific CAR-T cells	CD133, GD2, Muc1, CD117	1	NCT03356782	20	N/A	Ongoing, recruiting

These results suggest that anti-IGF-1R antibody treatment may provide therapeutic benefit for a select group of patients, but additional efforts are needed to identify biomarkers that can predict which subset of patients will respond. In the cixutumumab trial, tumor levels of IGF-1, IGF-2, and IGF-1R were evaluated by immunohistochemistry, but there was no correlation between expression of these three proteins and response to cixutumumab treatment (94). On the other hand, in the figitumumab trial, patients with intermediate pretreatment IGF-1 levels had improved survival compared to patients with lower baseline IGF-1 levels (93). Data from the R1507 trial suggest that high baseline IGF-1 levels correlate with improved overall survival but not with response to treatment (91). Taken together, IGF-1 levels may be prognostic but did not show consistent utility in predicting response to anti-IGF-IR therapy (111). In addition, given the relatively low response rates in the anti-IGF-1R clinical trials, it may be necessary to re-examine the mixed results of preclinical and clinical studies as well as the modest biological evidence underlying IGF-1R as a target for ES therapy. Specifically, more rigorous preclinical data may be needed to develop strategies in targeting the IGF family before further development of clinical trials.

Furthermore, mechanism-based molecular approaches utilizing combination strategies may prove more efficacious than IGF-1R antibody monotherapy. For example, the combination of IGF-1R antibodies with mTOR inhibitors has been evaluated in ES, with the rationale that mTOR inhibitors can induce AKT phosphorylation and signaling via an IGF-1R dependent mechanism; (112) given this dependence, it was thought that the combination of an IGF-1R antibody and mTOR inhibitor would have the potential to overcome the resistance seen when either was given as monotherapy. For ES specifically, the combination of ganitumab with rapamycin showed efficacy in preclinical modes (113), and the combination of cixutumumab and temsirolimus in the phase I setting for patients with refractory ES showed durable tumor regression in 29% of patients (114). However, a subsequent phase II study evaluating the efficacy of cixutumumab and temsirolimus among 46 patients with refractory or recurrent pediatric sarcoma (12 of whom had ES) showed no objective responses (96).

### Other Tyrosine Kinases

Besides IGF-1R, other receptor tyrosine kinases (RTKs) active in ES have been explored as potential therapeutic targets given the key role of RTKs in tumor growth and survival, and the success of RTK-inhibitors in other cancers. For example, c-KIT and platelet-derived growth factor receptor β are both expressed in ES, and treatment of ES with imatinib (which inhibits phosphorylation of KIT and platelet-derived growth factor receptors) results in decreased proliferation and enhanced antitumor activity of both ES cell lines (115) and xenografts (116). However, three phase II trials treating patients with ES with imatinib showed either no response in all ES patients enrolled (102) or partial response only in one patient (103, 104).

Epithelial growth factor receptor (EGFR) inhibition has also been explored in preclinical models of ES, showing decreased cell growth with high doses of gefintib *in vitro* (117), but minimal activity *in vivo* (118). Vascular endothelial growth factor (VEGF) inhibition similarly results in decreased cell growth as well as reduced tumor vessel density in preclinical models (119–121). Bevacizumab (monoclonal antibody targeting the VEGF receptor), has been tested in a phase II study through the COG including seven patients with ES (four of whom completed therapy), with results pending (NCT00516295). Similarly, pazopanib (multi-kinase inhibitor with activity against VEGF) was tested in a phase I study of children with soft tissue sarcoma (including three patients with ES), showing that it was well-tolerated with evidence of anti-angiogenic effects (105). However, as ES is not dependent on the EGFR and VEGF pathways for oncogenesis and proliferation, it is unclear how much patients with refractory disease will ultimately benefit from targeting of these pathways.

Potratz et al. found that nine individual RTKs were more active in ES tumors derived from metastatic disease than localized disease (122). Among these 9 RTKs, the authors further explored the role of ROR1 in ES given its promising results as a therapeutic target in leukemia (123) and metastatic carcinomas in preclinical models (124, 125). The authors showed that silencing of ROR1 resulted in dysfunctional migration of ES cells *in vitro*, with the conclusion that ROR1 may also be a potential therapeutic target for ES. A second study found that high expression of the RTK, AXL, was a significant predictor of poor survival, and that inhibition of AXL with BGB324 resulted in decreased cell growth, viability, and migratory capabilities of ES cells *in vitro* (126). The authors concluded that AXL is also a potential target for ES.

Spleen tyrosine kinase (SYK) is a non-receptor tyrosine kinase that may also serve as a targetable oncogene in ES. SYK is known to promote cell survival in a variety of pediatric tumors including leukemia and retinoblastoma (127). Sun et al. recently found that SYK is also highly phosphorylated and active in ES, with inhibition of SYK resulting in decreased cell growth both *in vivo* and *in vitro* (128). This study also identified c-MYC as an SYK-promoted gene that in turn could activate transcription of MALAT1, resulting in tumor growth. Given the oncogenicity that results from activation of the SYK/c-MYC/MALAT1 pathway, inhibition of SYK signaling may be a potential treatment strategy for ES, although further preclinical studies testing this hypothesis are needed before translation into the clinical setting.

### TRAIL

Tumor necrosis factor-related-apoptosis-inducing ligand (TRAIL) is a member of the tumor necrosis factor (TNF) family that plays a key role in immunosurveillance and apoptosis. The binding of TRAIL to death receptors (TRAIL-R1 and TRAIL-R2) leads to the activation of the extrinsic apoptosis pathway (129). Pediatric soft tissue sarcomas including ES and rhabdomyosarcoma are sensitive to TRAIL-induced apoptosis (130, 131). As TRAIL-R1 and TRAIL-R2 have restricted expression on normal tissue, TRAIL receptors are attractive immune targets. A monoclonal antibody activating TRAIL-R2, lexatumumab, has been tested in the phase I setting for adult solid tumors (132), as well as pediatric solids tumors including four patients with ES, with results showing some anti-tumor activity but no partial or complete responses (106).

### Gangliosides

The ganglioside, GD2, is a cell-surface molecule with a highly restricted pattern of expression, found in neuroectoderm-derived tumors and sarcomas, including ES. Although the expression level of GD2 is heterogeneous across different ES cell lines and primary ES cell cultures, GD2 is still a potential cell surface target for treating ES, with expression levels ranging from 40 to 90% from diagnostic biopsy samples (133, 134). In addition, anti-GD2 antibodies have been actively tested in clinical trials for neuroblastoma for over two decades, with proven safety and efficacy (23, 24, 135–137). An ongoing clinical trial study at St. Jude is testing the role of the anti-GD2 antibody, hu14.18K322A, in the treatment of ES (NCT02159443). GD2 remains an attractive target in ES, but more preclinical and clinical data are needed to justify this approach.

GD3 is a ganglioside that is primarily expressed on human melanoma tissues (138), with recent findings of high expression levels on pediatric tumors including osteosarcoma, ES, rhabdomyosarcoma, and desmoplastic small round cell tumor (DSRCT) (134). Although there are no randomized trials testing anti-GD3 antibodies as there are for GD2, the targeting of GD3 has shown activity in phase I trials including patients with melanoma (139, 140). In addition, a phase I trial of a bivalent GD2/GD3 vaccine for neuroblastoma showed encouraging survival benefit (141), with a phase II study ongoing. Like GD2, GD3's expression is largely limited to malignant cells and some activated T cells, making it a potential immune target for ES. N-glycolated GD3 (Neu-Gc-GM3) also has a restricted expression pattern on malignant cells and not normal tissues. A controlled phase II trial in patients with metastasis breast cancer with a Neu-Gc-GM3 based vaccine showed that the vaccine was well-tolerated, immunogenic, and had encouraging efficacy (142). Neu-Gc-GM3 is also expressed on the surface of ES, Wilm's tumor, and neuroblastoma (143), making it another potential target for treatment of ES that could avoid non-specific toxicity.

### B7-H3

B7-H3 is a cell surface immunomodulatory glycoprotein that could play a role in tumor progression via the inhibition of T cells and natural killer cells (144). B7-H3 is overexpressed in a variety of adult and pediatric tumors including ES (145), and shown to be a good target for tumor purging before stem cell transplant (146). Radioimmunotherapy directed at B7-H3 using the antibody, 8H9, has been tested in the phase I setting for patients with DSCRT (NCT01099644), neuroblastoma with central nervous involvement (NCT00089245 and NCT03275402), and diffuse intrinsic pontine glioma (NCT01502917). Results have shown minimal toxicity and encouraging efficacy, with the potential to increase survival in patient populations that have very few treatment options (147–149).

### Endosialin

Endosialin (also known as tumor endothelial marker-1 or TEM-1) is a cell surface glycoprotein that is found on mural cells, myofibroblasts, as well as a variety of pediatric tumors including ES, rhabdomyosarcoma, osteosarcoma, synovial sarcoma, and neuroblastoma (150–153). Endosialin promotes tumor cell growth and neovascular formation via the platelet-derived growth factor (PDGF) pathway (154). Ontuxizumab is a humanized monoclonal antibody targeting endosialin and has the ability to block PDGF signaling and tumor stroma organization (155). A phase I study of ontuxizumab in relapsed or refractory pediatric solid tumors (including four patients with ES) showed that ontuxizumab was well tolerated, although no objective responses were seen (107).

### STEAP1

Another potential therapeutic target for ES includes the six-transmembrane epithelial antigen of the prostate 1 (STEAP 1). STEAP1 is a 339-amino-acid protein named for its six transmembrane spanning regions, and is upregulated in a variety of tumors, including prostate, bladder, ovarian, rhabdomyosarcoma, and ES (156, 157). Grunewald et al. utilized transcriptome and proteome analyses as well as functional studies to show that STEAP1 expression correlates with oxidative stress responses and elevated levels of reactive oxygen species. This in turn regulates redox-sensitive and pro-invasive genes, suggesting that STEAP1 may be associated with an invasive phenotype of ES (158). Grunewald et al. also found that STEAP1 can serve as an immunohistological marker for patients with ES; 71 of 114 (62.3%) ES samples displayed detectable membranous STEAP1 immunoreactivity, making STEAP1 a potential therapeutic target (159). Another genetic profiling study done in ES patients showed that the absence of STEAP1 transcript in the bone marrow was strongly correlated with patient overall survival and survival without new metastases (160). Given the expression of STEAP1 in >60% of ES tumors but with limited expression in normal tissue (secretory tissue of the bladder and prostate) (161, 162), it could be a useful target for antibody-based and T-cell based strategies.

### CD99

CD99 antigen, also known as MIC2 or single-chain type-1 glycoprotein, is a heavily O-glycosylated transmembrane protein with a molecular weight of 32 kD. It is expressed on leukocytes and is believed to increase T-cell adhesion and function in apoptosis (88, 163). CD99 is also expressed on the surface of ES cells, making it an attractive tumor target. Reduced CD99 expression leads to neural differentiation of ES cells, suggesting that CD99 may have role in the inhibition of neural differentiation (164). In addition, knockdown of CD99 in ES cell lines results in decreased oncogenic potential, including decreased growth in tissue culture, diminished colony formation in soft agar assays, reduced cell motility, and smaller tumors with less metastasis in xenograft models. Given CD99's involvement in apoptosis, CD99 engagement in ES cell lines have led to caspase-independent cell death (165, 166), making anti-CD99 antibody therapy another attractive therapeutic approach (75). Cu-labeled anti-CD99 antibodies were shown to be superior to FDG-PET in detecting micrometastases in xenograft models (167). A combination of doxorubicin and an anti-CD99 antibody could improve mouse survival (168, 169). Unfortunately, CD99 is not only expressed on ES but also on normal human tissues including the testis, gastric mucosa, prostate, and hematopoietic tissues, with potential of off-tumor, on-target bystander toxicities when used in humans (170).

### Methionine Depletion

Cancer cells require methionine for aberrant transmethylation. As a result, cancers develop a dependence on methionine (171), with deprivation of methionine resulting in cell cycle arrest and eventually apoptosis (172). Recombinant methioninase (L-methionine-cleaving enzyme from *Pseudomonas putida)* acts to deplete methionine and in a variety of tumors including ES, results in arrested cell growth in preclinical models (typically at the S/G2 phase) (173). Although prolonged use of recombinant methioninase is not feasible given the potential liver toxicity, there has been interest in combining recombinant methioninase with standard chemotherapeutic agents, especially those active in S/G2 (171). For example, in preclinical models of neuroblastoma, recombinant methioninase showed synergism with microtubule depolymerization agents (174), and in preclinical models of synovial sarcoma, overcame resistance to doxorubicin monotherapy (175).

## Immunotherapy

Immunotherapy is a treatment modality for many human solid tumors. Unfortunately, ES belongs to the majority class called “cold” tumors where little immune and/or inflammatory infiltrates are present, whether as a result of immune privilege, immune escape, or immune inhibition by the tumor microenvironment. Consistent with the absence of tumor infiltrating lymphocytes (TILs), ES tumors typically have low expressions of immune checkpoint molecules including PD-1 and PD-L1 (176). Nevertheless, the observation of an increased number of tumor-infiltrating CD8+ T cells associated with decreased tumor progression of ES might suggest a role for T cell based therapy if only T cells can be educated appropriately (177).

### Cell-Based Immunotherapy and Vaccines

Cell-mediated immunotherapy strategies explored in ES include both T cell cancer vaccines and cell-based immunotherapy. Peptides produced from the EWS-FLI1 fusion protein are weakly immunogenic and do not significantly stimulate cytotoxic T-lymphocytes (CTL). Methods to enhance antigen presentation and strengthen the immunogenicity of the EWS-FLI1 peptides have been explored. For example, Evans et al. found that a modified peptide, YLNPSVDSV, induced strong CTL killing in ES cells, and the adoptive transfer of these specific CTLs into mice killed ES xenografts and increased survival (178). A translocation specific peptide vaccine has also been tested a pilot study in humans, although disappointingly with no impact on patient outcomes (179). Membrane-associated phospholipase A1 beta (LIPI) is a cancer/testis antigens (CTA) that is highly tumor specific, making it a potential target for immune-based therapies as well (180). Mahlendorf et al. found that CTLs targeting LIPI-derived peptides, LDYTDAKFV and NLLKHGASL, were able to kill ES cells *in vitro* (181).

Ghisoli et al. tested the efficacy and safety of a therapeutic vaccine known as FANG immunotherapy, consisting of autologous tumor cells that have been transfected with RNAi bi-shRNA furin and the rhGMCSF transgene. This creates a vaccine that assists in antigen presentation and the recruiting of regional nodal migration of dendritic cells (via GM-CSF), with the possibility of negating the immunosuppressive proteins including TGB1 and TGB2 (via furin). A pilot trial of FANG immunotherapy given to 12 patients with advanced or metastatic ES showed a good safety profile and successful elicitation of a tumor-specific systemic immune response in all patients. Additionally, the 1-year overall survival for this heavily pre-treated cohort was 75% (182). A two-part Phase II study utilizing FANG immunotherapy in patients with refractory ES is now ongoing (NCT02511132). In the first part, patients with refractory ES are randomized to either FANG immunotherapy or chemotherapy with gemcitabine and docetaxel; while in part two, patients with refractory ES receive FANG immunotherapy in combination with irinotecan and temozolomide. Another strategy utilizing dendritic cell vaccination with or without recombinant human IL7 has been tested in patients with metastatic and recurrent ES. The 5-year survival in the intent-to-treat analysis for patients with newly metastatic ES or rhabdomyosarcoma was 77%, with a T cell response to autologous tumor lysate seen in 62% of patients (183).

Natural killer (NK) cell based immunotherapy is another potential treatment strategy for ES. In the absence of tumor specific antibody, NK cells can kill ES tumors. In the presence of specific antibodies, NK cells mediate efficient antibody-dependent cell mediated cytotoxicity (ADCC). Cho et al. tested NK cell cytotoxicity in a variety of pediatric tumors, and found that ES cells are sensitive to the cytotoxicity of expanded, activated NK cells *in vitro* (184). NK cell killing of ES cells is specifically mediated via NKG2D and DNAM-1 receptor dependent pathways (185), and histone deacetylase inhibitors can enhance expression of NKG2D ligands and increase the sensitivity of ES cell lines to NKG2D-depedent toxicity (186). Additionally, expanded NK cells have shown efficacy in treating immunodeficient mice with ES tumors, resulting in long-lasting disease control (184).

### Immune Checkpoint Inhibitors and CAR-T Cells

Immune checkpoint inhibitors have been evaluated in a multitude of clinical trials involving many different cancer types and are currently an area of active investigation in ES (Table 2). Ongoing clinical trials testing checkpoint inhibitors in ES patients include: Ipilimumab (anti-CTLA4, NCT02304458), Nivolumab (Anti-PD1, NCT03190174), Ipilimumab + Nivolumab (NCT02982486), and Enoblituzumab (B7-H3, NCT02982941). Given the paucity of mutations in ES (hence low frequency of neoantigens) accompanied by low expression levels of PD-1 and PD-L1, it remains questionable if patients with ES can derive significant clinical benefit from these agents.

To overcome the low frequency of tumor-specific T cell clones, CAR T cells may provide an alternative option. In one adoptive T cell study, patients are randomized to either EGFR-specific CAR T cells or CAR T cells directed at both EGFR and CD19 (NCT03618381). A second ongoing trial is treating patients with relapsed or metastatic ES with 4th generation CAR T cells (NCT03356782). Another strategy explored in a pilot study of ES patients utilized chondromodulin-I/HLA-A^*^02:01/antigen-specific allorestricted T cells, with a treatment response seen in one of three patients (187). Given the lack of success of CART across a broad spectrum of human solid tumors, more research is probably needed before the success of CD19 CART could be translated into solid tumor systems. Their clinical application will likely have to wait for more in-depth research.

## Future Directions for Combined Modality Treatment

Although the novel therapies discussed above have shown encouraging results in preclinical models of ES, successful integration into the clinical setting remains challenging. Careful consideration must be given to the timing of signaling blockade (with small molecules) and immunotherapy (whether antibody-based or cell-based) in relation to standard chemoradiation in order to maximize clinical benefit. For example, patients with locally advanced non-small cell lung cancer treated on the PACIFIC trial were randomized to receive either placebo or adjuvant durvalumab (PD-L1 inhibitor) after completion of definitive chemoradiation (188). The addition of adjuvant durvalumab significantly improved progression-free survival, with the benefit exclusively seen in patients who received durvalumab within 2 weeks of completion of RT. These findings highlight the importance of the timing of immunotherapy in relation to RT. However, the ideal sequencing of immunotherapy with standard therapy for many adult solid tumors remains unknown, and preclinical data have largely resulted in conflicting data (189). Similarly how the inhibition of signaling pathways could be optimally combined with cytotoxic therapy, either concurrently or sequentially, requires more detailed testing in preclinical models. Perhaps combination therapy targeting multiple signaling pathways to overcome heterogeneity and resistance is one of several principles one can borrow from the past experience in cancer therapeutics.

In ES, where the intensive induction chemotherapy is immunosuppressive, a 6–12 month recovery period is generally needed for any adaptive immunity to be operational (190). As in metastatic neuroblastoma treated with N6/N6-like induction regimens (from which P6 regimen was derived), a viable immunotherapy option immediately post-chemotherapy is the “passive” monoclonal antibody approach (e.g., against GD2). That success in neuroblastoma is predicated on its sensitivity to myeloid-ADCC and cell-mediated cytotoxicity, where the effector cells and proteins (neutrophils and complement) can rapidly recover even after strong chemotherapy, and where myeloid cells can be put into overdrive using growth factors such as GM-CSF. On the other hand, even though CD16(+) NK cells that mediate NK-ADCC recover faster than T and B cells, their numbers are still suboptimal. Furthermore, after many years of testing, IL2 is now proven to have no role in accelerating their recovery or function to impact patient outcome (191). For neuroblastoma, the 6-month immune convalescence has pushed the timing of the GD2/GD3 vaccine to later on during consolidation when patients could make a meaningful anti-ganglioside immune response. It is expected that T cell based therapies using bispecific antibodies (BsAb), T cell vaccines, or checkpoint inhibitors will likely require a similar recovery period. Alternatively, if healthy autologous T cells are cryopreserved before significant chemotherapy damage, or if third party antigen-primed (e.g., EBV) T cells, can be activated *ex vivo* for arming with BsAb or for viral transduction to make CARTs, they may be ideal for “passive” adoptive cell therapy. KIR-mismatched NK cells may also be used for cell therapy with or without anti-tumor antibodies, although their expansion *ex vivo* will require cocktails of interleukins (e.g., IL7, IL15, IL21).

For patients with locally advanced ES, administration of “passive” immunotherapy (using tumor-selective antibodies, CART, BsAb, or BsAb armed-T cells), either integrated with or right after the completion of definitive radiation therapy (as done on the PACIFIC trial) may provide the most benefit given the immunomodulatory effects of RT such as increased neoantigen presentation and enhancement of T-cell infiltration (192–195). Again, full immune recovery will not be complete for at least 6–12 months, at which point a vaccine program may be most useful. For patients with metastatic disease, adding immunotherapy to chemotherapies that are not immunosuppressive could be advocated (196–198). Such combination strategies of immunotherapy with RT have been explored in many preclinical and clinical studies of adult solid tumors (195, 198). However, parallel studies in pediatric solid tumors including ES remain largely unexplored. A more in-depth immune profiling and genomic tracing of ES as patients recover from induction chemoradiotherapy, or suffer relapse, is critically important to inform the future design and integration of immunotherapy and small molecules into the standard of care of ES.

Further consideration must also be taken to incorporating ongoing discoveries of the molecularly diverse underpinnings of ES. For many years, the heterogenous clinical presentations and outcomes of ES were at odds with its relative genomic homogeneity, consisting of near-universal *EWS-FLI1* fusion alterations and relatively few other recurrent somatic alterations (199). However, recent in-depth epigenomic profiling of ES has uncovered significant inter-individual and intra-tumoral heterogeneity of DNA methylation states, most pronounced in metastatic tumors (200). Additionally, large genomic sequencing efforts in ES have shown that the presence of intra-tumor genetic heterogeneity at diagnosis affects the evolution of recurrent ES tumors, with an ~3-fold increase in number of genetic alterations seen in relapsed samples (199). This genetic and epigenetic intra-tumoral heterogeneity likely plays a substantial role in driving clonal evolution and clinical response to therapy (201). Therefore, incorporating in-depth molecular profiling both at diagnosis and at the time of recurrence may be imperative to the potential design and success of precision-targeted ES therapy.

## Conclusion

Proteomics and genomic analysis of normal and tumor tissue have led to the discovery of many genes whose downstream products may provide substrates for targeted therapies. Valuable insights into the role of different targets in ES biology have been gained throughout the past two decades. Antibody-based and cell-based immunotherapy have emerged rapidly as potential modalities for ES (20). Many have shown promising results in preclinical models. The clinical success of GD2-based immunotherapy in neuroblastoma could provide a framework in designing the next generation strategies for metastatic ES, a tumor much less lethal than high risk neuroblastoma historically. The successful integration of biologic targeted therapies and immunotherapy into standard of care will be the future challenge in changing the natural history of a lethal disease.

## Author Contributions

DC, T-YL, and N-KC contributed to the conception and design of the review and wrote sections of the manuscript. DC wrote the first draft of the manuscript. All authors contributed to manuscript revision, read, and approved the submitted version.

### Conflict of Interest Statement

N-KC reports receiving commercial research grants from Y-mabs Therapeutics and Abpro-Labs Inc.; holding ownership interest/equity/options in Y-Mabs Therapeutics Inc., and in Abpro-Labs, and owning stock options in Eureka Therapeutics. N-KC is the inventor of pending and issued patents filed by MSK, including hu3F8 and 8H9 licensed to Ymabs Therapeutics, beta-glucan to Biotec Pharmacon, and HER2 bispecific antibody to Abpro-labs. N-KC is an advisory board member for Abpro-Labs and Eureka Therapeutics. The remaining authors declare that the research was conducted in the absence of any commercial or financial relationships that could be construed as a potential conflict of interest.
